# 

**Published:** 1889-06-08

**Authors:** 


No. 141, Vol. VI.—June 8th, 1889

[Registered at the General Post Office as a Newspaper.]

Per Annum, 6s. 6d., post free.
Monthly Parts, 6d.; post free, 7½d.
Ditto per Annum, 7s. 6d., post free.

